# Experiences and challenges of using community health worker-led mechanism in supporting HIV disclosure among adults living with HIV in heterosexual relationships in the rural Uganda

**DOI:** 10.1186/s12981-023-00508-0

**Published:** 2023-03-11

**Authors:** Zubair Lukyamuzi, Bashir Ssuna, Ruth Nabisere Mirembe, Denis Mawanda, Paul Kinkumu, Christine Nalugo, Esther Adikin, Faridah Namisango, Rita Nakalega, Patience Atuhaire, Philippa Musoke, Lisa M. Butler

**Affiliations:** 1 Johns Hopkins University Collaboration (MU-JHU), Makerere University, Upper Mulago Hill Road, Kampala, Uganda; 2 School of Public Health, Makerere University College of Health Sciences, Kampala, Uganda; 3 Uganda Tuberculosis Implementation Research Consortium (U-TIRC), Kampala, Uganda; 4 Infectious Diseases Institute (IDI), College of Health Sciences, Makerere University, Kampala, Uganda; 5 Mildmay Hospital and Institute of Health Sciences, Mildmay Uganda, Box 24985, Kampala, Uganda; 6 Islamic University in Uganda, Box 2555, Mbale, Uganda; 7 Institute for Collaboration On Health, Intervention, and Policy, University of Connecticut, Storrs, CT 06269 USA

**Keywords:** HIV disclosure, Heterosexual partners, Community health workers, Experiences, Challenges

## Abstract

**Background:**

HIV status disclosure among sexual partners is vital in HIV management. Community health workers (CHW) support HIV disclosure among adults living with HIV (ALHIV) in sexual relationships with disclosure difficulties. However, experiences and challenges of using CHW-led disclosure support mechanism were not documented. This study explored experiences and challenges involved in using CHW-led disclosure support mechanism among ALHIV in heterosexual relationships in the rural Uganda.

**Methods:**

This was a phenomenological qualitative study involving in-depth interviews among CHWs and ALHIV with HIV disclosure difficulties to sexual partners in greater Luwero region, Uganda. We conducted 27 interviews among purposively selected CHWs and participants who had participated in the CHW-led disclosure support mechanism. Interviews were conducted until saturation was reached; and analysis was done using inductive and deductive content analysis in Atlas.

**Results:**

All respondents viewed HIV disclosure as an important strategy in HIV management. Provision of adequate counseling and support to those intending to disclose was instrumental for successful disclosure. However, fear of the negative disclosure outcomes was viewed as a barrier to disclosure. The CHWs were viewed as having an added advantage in supporting disclosure as opposed to the routine disclosure counseling. However, HIV disclosure using CHW-led support mechanism would be limited by possible bleach of client’s confidentiality. Therefore, respondents thought that appropriate selection of CHWs would improve their trust in the community. Additionally, providing CHWs with adequate training and facilitation during the disclosure support mechanism was viewed to improve their work.

**Conclusion:**

Community health workers were viewed as being more supportive in HIV disclosure among ALHIV with disclosure difficulties to sexual partners compared to routine facility based disclosure counseling. Therefore, near location CHW-led disclosure mechanism was acceptable and useful in supporting HIV disclosure among HIV-affected sexual partners in rural settings.

## Introduction

Human Immunodeficiency Virus (HIV) remains a public health problem worldwide, with 650,000 AIDS-related deaths and 1.5 million new infections occurring in 2021; [1, 2], and sub-Saharan Africa remains the most affected region [3, 4]. Despite HIV affecting all age groups, sexually active people are the most affected. In Uganda, about 10% of sexual couples are affected by HIV, with only 3% being concordant positive [4]. In the HIV management, non-disclosure of HIV status among sexual partners remains a critical challenge [5, 6] due to its association with poor HIV management outcomes, including unsuppressed viral load due to poor ART adherence, development of resistant strains, and increased HIV transmission [7–9]. In contrast, disclosure promotes desirable HIV management outcomes, social support, trustworthiness, and social acceptance [10–13].

In a sexual relationship, HIV disclosure is influenced by various factors, including the type of the relationship and bio-behavioral and socioeconomic factors [12, 14, 15]. Specifically, these factors include literacy, economic dependence, number of sexual partners, place of index HIV testing, ART status, receipt of disclosure counseling, time spent with HIV, perceived stigma, social desirability bias, and others [11–13, 16–19]. Consequently, disclosure can be associated with negative outcomes such as blame, abandonment, and separation [20, 21]. HIV status disclosure can be done by the people living with HIV (PLHIV) themselves or by other people such as health workers on behalf of the PLHIV following their consent [16]. Additionally, disclosure is a process and not a one-time event [20], and may follow a planned behavior change model, including the theory of planned behavior change [21–23].

Despite its fundamental role in the HIV management [24, 25], limited interventions are available to support disclosure, especially among PLHIV in sexual relationships with disclosure difficulties. The use of a professional facilitated couple counseling approach by healthcare workers has shown to be effective in enhancing disclosure [26]. However, the approach is limited by the inadequate number of trained health professionals in low and middle-income countries (LMIC) [27, 28]. To minimize the gap for the scarcity of trained health professionals in LMICs, the Community Health Worker (CHW) program re-emerged as a desired strategy to increase access to health services [29–31]. Uganda adopted the CHW strategy in 2001, and currently almost all villages in the country have at least one CHW serving at least 5–10 households [32]. Additionally, the approach was also thought to address African men’s reluctance in seeking HIV care services, including HIV-related services [33].

The contribution of CHWs in improving access to HIV care has been documented [34–36]. Moreover, CHWs’ role in supporting HIV status disclosure among PLHIV in sexual relationships was also reported [37]. However, the experiences of using the CHW-led disclosure support mechanism were not documented. Therefore, this study aimed to explore experiences and challenges of using CHWs in supporting disclosure among HIV-affected sexual partners in the greater Luwero region.

## Methods

### Study design

The methods of the current study were part of the primary study which aimed to assess the effect of CHWs in supporting the HIV disclosure among ALHIV in heterosexual relationships in the greater Luwero region [37]. The primary study was a quasi-experimental design with two study arms allocated by clusters. Clusters were sub-counties of the greater Luwero region in Uganda, which had been previously determined according to the geographical boundaries. From October 3rd, 2019, to May 31st, 2020, the main study was conducted among ALHIV and CHWs. Participants from geographically close clusters were allocated either the intervention (CHW support) or control arm (without CHW support). The intervention and control clusters were separated by a geographical barrier (buffer zone) to minimize cross-contamination. All potential participants from the buffer zone were excluded from the study. The current sub-study was a phenomenological research design that involved qualitative interviews among participants and CHWs who were involved in the above primary study.

### Study area and population

The study area was the greater Luwero region, located about 20 km from Kampala, the capital of Uganda, which together with the study population were described elsewhere [37].

Participants in the study were ALHIV and CHWs. ALHIV were those who had been in heterosexual relationships for at least three months and had not disclosed their status to their current primary partners. CHW were those who were coming from the same residence as participants (ALHIV) and were fully registered in the district CHW registry. Community health workers provided HIV disclosure support to the participants allocated to the intervention arm.

### Study interventions

The study arms which included the CHW intervention arm and control arm (routine care) were described else [37].

### Outcomes

The outcomes of this sub-study were the experiences and challenges of using CHWs in supporting HIV disclosure among ALHIV in heterosexual relationships.

## Sample size and sampling procedures

In the current qualitative sub-study, we purposively selected participants and CHWs for in-depth interviews. Respondents were sampled for the interviews until saturation was reached. In total, 13 and 14 interviews were conducted among CHWs and participants, respectively.

### Data collection procedures

Participants and CHWs of the current study were recruited between 3rd October 2019 and 7th November 2019 as part of the main study following the eligibility criteria which were described elsewhere [37]. Respondents for the interviews were purposively selected, particularly those; who had used the CHW-led disclosure support mechanism. Respondents were also selected according to the outcomes of the disclosure process and the challenges faced, which were informed by the main study. CHW respondents were also purposively selected according to their experience in the community health, number of disclosures supported, and challenges faced. All interviews were conducted at the study sites by a trained social scientist (facilitator) and the corresponding author (note taker) in the participant’s or CHW’s preferred language. All interviews were conducted in private rooms where conversations could not be overheard. Interviews were about 45 min long and were audio-recorded with the participant’s or CHW’s permission.

### Theoretical framework

To thematically categorize the experiences and challenges, we followed the Theory of Planned Behavioral (TPB) change which postulates that expectation or belief in significant others and the availability of an enabling environment are important constructs in behavior change [22, 38]. CHWs facilitated disclosure, and this provided an enabling environment. According to the TPB, three factors determine behavior change: (a) attitudes, (b) subjective norms, and (c) perceived behavioral control [39], as shown in Table 1. Therefore, CHWs instilled a positive attitude towards disclosure in the PLHIV. CHWs and participants’ regular contact through phone calls and home visits probably formed a subjective norm or standard that was adhered to by participants, and this reduced HIV-related stigma. The CHW-led disclosure support mechanism also brought disclosure services closer to the PLHIV in the community.

**Table 1:** Application of the theory of planned behavioral change constructs to HIV status disclosure among HIV affected heterosexual sexual partners

Construct	Application
Attitudes	Community Health workers counselled participants about HIV disclosure in a sexual relationship. This improved the participant’s understanding of HIV and the importance of disclosure, hence instilling positive attitudes toward HIV disclosure among participants
Subjective or standard norms	During disclosure support, CHW counseled, home visited, and telephoned participants on a regular basis. This was thought to have become a standard norm of interaction between CHW and participants. This standard norm increased self-efficacy and confidence among participants, hence gaining strength to disclose
Perceived behavioral control	Community-based HIV disclosure support by CHW brought disclosure services close to the PLHIV in their communities, which increased the perceived control of their disclosure behavior

### Data analysis

We intended to gain a deeper understanding of the experiences, enablers, barriers, and challenges involved in using the CHW-led disclosure mechanism. A total of 27 interviews were conducted: 14 among participants and 13 among CHWs.

Audio recordings were transcribed verbatim directly into English by the study team within one week of data collection. Quality checks were performed for each transcript, and appropriate corrections and revisions were made. All interviews were analyzed for emergent themes using inductive and deductive content analysis approaches [40]. During the inductive analysis, open coding was carried out to identify specific portions of text. Provisional labels were defined and illustrated to become codes (Table 2), which were assembled into a codebook. Data were coded by two coders (social scientist and corresponding author) [41]. After the development of the initial codebook, we reviewed the results in the codebook for consistency of text segmentation and code application with the continued inter-coder agreement. The coders reached a consensus and grouped identified codes into themes after reviewing inconsistent codes. The Consolidated Criteria for Reporting Qualitative Studies checklist was used to report study findings [41].

**Table 2:** Examples of meaning units, codes, categories, and themes from the interview content analysis among CHW and participants involved in the CHW-led disclosure mechanism in the rural Uganda

Meaning units/quotes	Codes	Category	Theme
“Telling my partner my HIV positive status, would help both of us because if I know that I am positive, and my partner is also positive, we can both take ARVs freely and this may help us prolong our lives.”	Adherence to HIV treatment to prolong life and care	Disclosure is done to improve adherence to HIV care	Disclosure facilitates adherence to HIV management strategies
“…that person or couple I am going to support in the disclosure process, I first make them my friends before we start any other discussions	Friendship with the a person living with HIV eases CHW-led disclosure mechanism	Client-CHW friendship facilitates disclosure when using CHW-led disclosure mechanism	Friendly relations between a client and the CHW motivate disclosure during CHW-led disclosure mechanism
“…they are very helpful especially during counseling. They counsel you and you feel like you can do it. And when you listen very well and do what you agree with them, you can easily disclose”	Adequate client counseling during the CHW-led disclosure mechanism	Attitudes towards the counseling offered by the CHW during the CHW-led disclosure mechanism	Positive counselling attitude in CHW-led disclosure mechanism motivates disclosure
“The advantage of using CHW in disclosure, issues like fights, arguments, separation may be minimized because a CHW may provide adequate and continuous counseling since they are always readily available to these couples.”	Fights, arguments, and separation reduce when the CHW-led disclosure mechanism is used	Negative disclosure outcomes reduce when the CHW-led disclosure mechanism is used	Un-eventful disclosure is achieved using CHW-led support mechanism

### Ethics approval and consent to participate

The protocol was reviewed and approved by the school of medicine Institutional Review Board (IRB)—Makerere University (REC REF 2019-100). Additional clearance was sought from the National council for science and technology (HS443ES). The district health departments granted permission to undertake the study. Informed written consent was obtained from all participants. Confidentiality and anonymity were strictly observed at all the research stages. All CHWs who were contacted and agreed to participate were trained on health ethics, confidentiality, and handling of adverse outcomes of disclosure. Additionally, we also obtained informed consent from the CHWs to participate in the study. Participant safety was ensured throughout the study. Participants who experienced adverse outcomes such as quarreling and separation were reconciled to the best of the study team’s ability before their termination from the study. Non-study partner HIV testing and referral to HIV care (for newly diagnosed positive non-study partners) were done upon their approval. All methods were carried out in accordance with the relevant guidelines and regulations of good clinical practice and human subject protection.

## Results

For the main study, a total of 245 participants were enrolled from 10 health facilities, with an average of 25 participants per facility. A total of 230 (93.9%) participants completed the study, and of these, 112 (48.7%) were in the intervention arm and 118 (51.3%) were in the control arm. The median age of participants was 30 (IQR = 25–37) years, and the majority were females, 176 (76.5%).

Forty-eight CHW aged between 25 and 60 were enrolled and all completed the study. The majority 40 (83.3%) were females.

Among the participants and CHWs of the main study, 14 interviews were conducted among participants and 13 interviews were conducted among CHWs for the current study, and the following were obtained.

### Disclosure of HIV status among participants

#### Importance of HIV status disclosure

Respondents viewed disclosure as an important strategy in the management of HIV. They mentioned that disclosure promotes adherence to HIV treatment and care, promotes HIV testing, prevents HIV transmission to a sexual partner, promotes psychosocial support, trust, and acceptance in a relationship. It also reduces negative outcomes that may result from one partner accidentally finding out another’s HIV positivity.

Telling my partner my HIV positive status, would help both of us because if I know that I am positive, and my partner is also positive, we can both take ARVs freely and this may help us prolong our lives. (Participant 010)

It also increases trust in a relationship if someone tells you the truth. I am sure my husband’s trust towards me increased following disclosure. (Participant 003)

Disclosure enables good adherence to ARVs. The partner disclosed to can also easily go and test for HIV; and if they are also positive, they can start ARVs early. Occasionally, if they test negative, they can support their HIV positive partner to take ARVs which can minimize the chances of HIV transmission. (CHW 004)

#### Enablers of CHW-led disclosure support mechanism

Some participants disclosed to their partners immediately after study entry because they received adequate assurance and counselling from the CHWs, healthcare workers, and study team.

The CHW first counseled me to be firm. Then I agreed with her to first have couple counseling with my partner at home. She home visited us and counseled us about HIV before disclosing to my husband directly. We discussed potential outcomes of HIV testing results and my husband confessed to be okay with any outcome. (Participant 005)

The good relationships or friendship between the CHWs and the clients in the community also facilitated disclosure.

…that person or couple I am going to support in the disclosure process, I first make them my friends before we start any discussions. (CHW 008)

#### Barriers to CHW-led disclosure support mechanism

Fear of the negative outcomes of HIV disclosure, including relationship breakups and fighting was a barrier reported by most of the respondents. This was a similar finding to both men and women. Accusations of infidelity and promiscuous behavior following disclosure was thought to result in undesirable outcomes.

Yes, I really fear a lot. The fear of telling him is there especially how to start it. I fear that maybe he can leave me after telling him or do something else. (Participant 013)

Some respondents reported incidences of breakups:

So, after the CHW supporting me to tell him, he was initially calm but later told me to pack up my things and go. I also packed them and went home. But after like a week, he came for me; I think after testing and found that he was HIV negative. (Participant 006)

I told her that it was important to know if she was also HIV positive so that we can protect the young ones. But she instead decided to react badly and weirdly. Currently she left home with her children. I do not know what to do because it seems she even blocked my phone number. (Participant 010)

Women were reported to be the most affected by negative outcomes of disclosure.

But unfortunately, there some people who face some challenges especially women who test positive during antenatal. If they tell their husbands about this, the husbands are likely to beat them or even leave them when pregnant. (Participant 002)

Community Health Workers also emphasized the fear of negative outcomes among clients during the CHW-led disclosure support.

The biggest challenge when one discloses to the partner is when the person disclosed to turns out to be HIV negative. This usually brings misunderstandings in the family. (CHW-002)

Fear of negative outcomes delayed disclosure for some participants, and one of the participants who had not disclosed said that she needed more time before making the final decision.

Then I got this new partner who doesn’t know my status. I was thinking of disclosing to him but I felt needed more time. (Participant 007)

### Attitudes and experiences in CHW-led disclosure support mechanism

#### Attitudes

Participants applauded the CHW-led disclosure mechanism and were positive about it; they said that CHWs gave them enough time during the disclosure process, which helped them to understand the importance of disclosure, hence making informed decisions. The participants further said that, CHWs supported them with disclosure skills, which helped them handle some negative outcomes of disclosure. The CHWs were also protective against negative outcomes, and were available to the participants to provide continuous support even after disclosure.

...they are very helpful especially during counseling. They counsel you and you feel like you can do it. And when you listen very well and do what you agree with them, you can easily disclose. …and the good thing with using a CHW is that you can first discuss with them and make a disclosure plan. And CHWs have some knowledge and skills on how to handle some situations…. they can also counsel you as a couple and support you even after disclosure. (Participant 005)

...the truth is that the mechanism was very beneficial. CHWs were very helpful because if there is something you wanted to talk to them about, you could just go and talk to them. So they were readily available. They could even come and check on us after disclosure for continuous support. So they did a great job towards our lives. (Participant 001)

Community health workers also had positive experiences and attitudes from the assignment as they received positive feedback from the participants they supported. They said the task was worth undertaking because they felt proud and satisfied after supporting successful disclosures.

…still the man came and told me that am relaxed, because I have been leaving the drugs at the work place which was making me miss some doses sometimes, but after disclosure, I feel like my journey is going to be good. (CHW 009)

Now like the one I told you about; she was very happy because she used to swallow her drugs hiding but now swallows openly; currently the couple even remind each other about drug adherence. So, people are very happy with the mechanism and are very appreciative. (CHW 007)

Recently I got information that the couple I supported to disclose stood up in front of a congregation at the HIV care facility and gave an HIV disclosure testimony; and advised the audience to always disclose their HIV status. (CHW 004)

like the first couple I worked on, the man was very happy that we let him know the truth about the positive HIV status of his wife. Currently he even escorts his wife for HIV care, and sometimes goes to pick medication for his wife despite himself being HIV negative. (CHW 006)

### Experienced challenges in the CHW-led disclosure support mechanism

Community health workers had to be patient, calm, polite, and empathetic during the disclosure process since each participant had a different story regarding their sexual relationships. The participants needed to be handled on a case-by-case basis, and CHWs required to have a working plan while executing the exercise.

The thing I have learnt is that, we have different personalities and backgrounds, and there are some people who are difficult to handle and you can find them annoying if you are not patient. They can respond to you inappropriately, and you realize that if you cannot control yourself you end up abandoning the process. But when you calm down and get to understand that people are not the same and should be handled on a case-by-case basis, you complete your task successfully. Additionally, some people may start from a high tone, and when you instead calm down, they eventually also calm down. (CHW 012)

…what I learnt from that program is a need for planning, if you are going to do anything, you have to plan, and plan early. (CHW 002)

Community health workers also faced challenges of inappropriate physical addresses given by some of the participants. Some participants kept changing locations and providing wrong directions. CHWs also had inadequate facilitation in terms of transport. These challenges led to a delay in the completion of the disclosure process.

I have found ups and downs in this exercise, first of all some clients are not easy to locate due to the fact that some of them are renting, especially the youths. So, today you may find them renting here and after some time you find them elsewhere. And some keep changing telephone contacts. So, it may take you time to get them; but if you persist you can finally get them. (CHW 003)

The challenge I have met is that some clients give wrong directions; so, you keep moving here and there looking for the given location and you end up using up all the transport money, and remember we were not given much money for transport, and sometimes, you end up using your own money because you have to complete the task. You find some when they shifted and when you call them, someone else answers and tells you that it is a wrong number (laughs). (CHW 005)

#### Advantages of the CHW-led disclosure support mechanism

Respondents felt that the mechanism was client centered as it really addressed the needs of PLHIV with disclosure difficulties. The mechanism reduced negative outcomes such as fights and separation. This was because CHWs were regarded as significant others in the community.

The advantage of using a CHW in the disclosure process is that, issues like fights, arguments, and separation may be minimized. This is because a CHW may provide adequate and continuous counseling due to the fact the CHWs are always readily available in the community. (CHW 011)

The advantage in using CHWs is that sometimes clients fear to discuss their issues with the healthcare workers at the health facility. This is because a healthcare worker might not understand the context of the client’s life in the village. So, it is beneficial to use a CHW because they know the client’s life right from the community. (CHW 004)

The advantages involved in using a CHW is that, if disagreements occur following disclosure, the client can easily reach out the CHW for help so that the disagreements do not escalate to separation. (CHW 008)

#### Limitations of the CHW-led disclosure support mechanism

Respondents said that the mechanism may be limited by issues of breaching of confidentiality.

Sometimes clients may fear that, if I tell a CHW, he/she will tell other people and the whole village will know. (Participant 010)

...no, there is no any danger because they were chosen by people; we chose them because there are like our healthcare workers whom we trust. Like for our CHW I have told you about, we can send her for anything concerning our lives and she is very beneficial; and keeps secrets. (Participant 012)

Therefore, clients who would accept this mechanism always trust their CHWs, and believe that the CHWs will not breach their confidentiality.

#### Improving the CHW-led disclosure support mechanism

Respondents advocated for adequate facilitation in terms of transport, increasing the number of CHWs, continuous training, and provision of working tools such as umbrella and gumboots.

What would have been added to this program is to provide adequate transport facilitation because looking for the client may even take you 3 days or even a month. Remember the motorcycle riders may charge you Ugx 2,000/= ($0.5) for a short distance. (CHW 005)

…adding more transport in terms of facilitation so that it does not burden us so much. (CHW 009)

to make this program better: more facilitation in terms of transport is needed. Because sometimes you have a client who has accepted to disclose but requires a facility setting for disclosure to be accomplished. So, if you don’t have enough money on you to escort them to the health facility, you miss out on the chance. Also training both the CHWs and community is important. (CHW 011)

Respondents said that the training keeps them updated with new ideas of handling clients.

## Discussion

This qualitative study aimed to explore experiences and challenges of using CHWs in supporting disclosure among HIV-affected sexual partners, and we found that: HIV disclosure was generally viewed as an important strategy in fostering adherence to HIV treatment and care, adequate counseling and support was important in the disclosure process, and fear of the negative outcomes hindered disclosure. The CHW-led disclosure support mechanism was viewed to have advantages which included the provision of adequate interaction time between the client and CHW, which enabled appropriate planning, and hence minimized the occurrence of negative disclosure outcomes. However, the mechanism required CHWs to be patient and empathetic to the client as well as handle each client according to the context of their sexual relationship. The mechanism was viewed to be limited by possible breach of the client’s confidentiality and inadequate transport facilitation. The mechanism was viewed to be successful if CHWs are adequately trained and provided with working tools. The implications of these findings are as below:

HIV status disclosure in a sexual relationship was important in increasing adherence to HIV treatment, such as adherence to ART and clinic appointments; this was a similar finding in the previous studies [10–13]. Following disclosure, clients reported increased psychosocial, emotional, and financial support from the partner, which augmented adherence to HIV management interventions, as previously reported [42–44]. For example, after disclosure, some non-study partners escorted their partners to the HIV care facilities, and some collected drugs (ART) from the HIV care facilities on behalf of their partners, as previously reported [45] Therefore, HIV disclosure among sexual partners, should always be prioritized in HIV care.

Adequate interaction time between a participant and the CHW was instrumental in achieving positive outcomes disclosure. This was due to the fact that such interaction allowed adequate counseling and support to the participant, which enabled participants to make appropriate rational disclosure decisions, as previously reported [12, 45]. Additionally, adequate counseling allows proper understanding of the requirements of HIV management, including disclosure to a sexual partner and possible consequences of disclosure. Moreover, counseling and disclosure support increases the confidence, self-efficacy, and self-esteem of a person intending to disclose, as previously reported [46, 47]. In the current study, CHWs supported the entire disclosure process through counseling, regular phone calls, home visits, and escorting the couple to the health facility for HIV counseling and testing. Therefore, ALHIV in sexual relationships with disclosure difficulties should be adequately counselled and supported through the entire disclosure process.

The HIV disclosure was limited by fear of negative consequences or outcomes which was similar to previous reports [20, 21]. Unfavorable consequences such as blame, domestic violence, abandonment, and separation were reported to hinder HIV disclosure [45, 48–50]. Although these outcomes are anticipated before disclosure, a few of these were reported in the current study. Additionally, the majority of those negative outcomes, which occurred were resolved and settled shortly. This finding was similar to the previous studies where fewer than anticipated negative outcomes were reported [51, 52]. Therefore, clients intending to disclose should always be assured that there is better than harm in disclosure to a sexual partner.

In regards to community-based healthcare support, the CHW-led disclosure support mechanism had extra advantages when compared to the routine care. Previous studies have reported CHW being at an advantage in supporting basic health-related interventions and programs in the community [34–36, 53]. Therefore, the current study strengthened the evidence that CHWs are important in supporting sexual partner disclosure among ALHIV with disclosure difficulties, as previously reported [37]. In the current study, CHWs were beneficial because they dedicated adequate time to counseling and supporting the clients as opposed to routine care. Moreover, CHWs also provided continuous post-disclosure counseling and support, which further stabilize and assured the couples after disclosure. In regards to the provision of basic healthcare services in the community, CHWs have an added advantage due to a lower CHW-to-patient ratio compared to the professional healthcare worker-to-patient ratio, as previously reported [54–56]. Compared to partner disclosure by the client themselves, the CHW-led disclosure support mechanism had more advantages because CHWs had better HIV-related knowledge, counseling, and disclosure skills, and were viewed as significant and protective to the disclosing client. This kind of protection minimized the negative outcomes of disclosure. Therefore, using the knowledge and skills obtained from training [57–60], CHW may be in a better position to offer basic HIV care services.

The CHW-led disclosure support mechanism required the CHWs to be patient and empathetic to the clients. This was similar to the previously reported qualities of HIV counseling and care, where healthcare workers were encouraged to always be calm, exercise patience in handling patients, and be empathetic [61–63]. Exercising such quality counseling enables the client to know that the healthcare worker or CHW understands the client’s situation. The clients should also be handled on a case-by-case basis, as people go through different situations regarding family, social, and intimate relationships, as previously reported [64, 65]. Therefore, when supporting disclosure, CHWs should contextualize each client’s situation.

The CHW-led disclosure support mechanism was limited by possible breach of confidentiality of the client intending to disclose. Although this didn’t occur in the current study, CHWs and participants were mindful of this possibility. Similarly, previous reports indicated that community members were concerned about CHWs interfering with the confidentiality of patients during the execution of their work [60, 66, 67]. In the current study, it was revealed that the trust a client had in their CHW alleviated fears of breaching confidentiality. Therefore, participants allowed CHW disclosure support only if they trusted their CHW. This was similar to previous studies, which indicated that the trustworthiness of healthcare workers among their clients was considered critical for the successful uptake of health-related interventions [68–70]. Therefore, CHWs should always be keenly selected and continuously trained to increase their trust in the community, as previously reported [36, 71–73].

Providing adequate training, facilitation, and working tools like umbrellas was critical for a successful mechanism; this was in line with the Ugandan CHW operations manual [71]. Training is vital as it equips CHWs with knowledge about HIV and skills that can help them confront challenges faced during the execution of their work, as previously reported [57, 71, 73, 74]. Facilitating CHWs, such as providing adequate transport support, increases their effectiveness and efficiency, as previously reported [36, 71, 75]. Working tools such as umbrellas, gumboots, and branded T-shirts can motivate CHWs to work and help them overcome weather challenges as they traverse in the communities. The provision of such items was recommended previously [54, 71, 76]. Therefore, the CHW-led disclosure support mechanism needs adequate logistic support.

The strength of this study was that we trained CHWs before being attached to the clients, regularly supervised and facilitated them, which resulted in their efficiency and effectiveness. Additionally, we solicited feedback from both the CHWs and the participants regarding the use of the CHW-led disclosure support mechanism, which attracted diverse views and opinions from both ends. Providentially, we observed convergent views from the CHWs and clients. CHWs in the current study had been in HIV care for a long time, and others had worked in HIV care before; this caused them to have substantial knowledge, experience, and skills regarding HIV care and counseling. Our findings were limited by the lack of generalizability, especially in HIV care settings with inexperienced CHWs regarding HIV care.

## Conclusion

Community health workers were at an advantage to support disclosure in a community-based setting, as opposed to routine disclosure counseling at the facility. However, CHW should be keenly selected, facilitated, trained, and provided with working tools to execute this task. Therefore, in adopting a CHW-led disclosure support mechanism, HIV care programs should ensure appropriate measures in the selection, training, and facilitation of CHWs.

## Data Availability

The transcripts used and/or analyzed during the current study are available from the corresponding author on reasonable request.
